# Comparative transcriptome analyses on silk glands of six silkmoths imply the genetic basis of silk structure and coloration

**DOI:** 10.1186/s12864-015-1420-9

**Published:** 2015-03-17

**Authors:** Yang Dong, Fangyin Dai, Yandong Ren, Hui Liu, Lei Chen, Pengcheng Yang, Yanqun Liu, Xin Li, Wen Wang, Hui Xiang

**Affiliations:** Kunming University of Science and Technology, 727 South Jingming Road, Chenggong District, Kunming, Yunnan Province 650500 China; State Key Laboratory of Genetic Resources and Evolution, Kunming Institute of Zoology, Chinese Academy of Sciences, 32 East Jiaochang Road, Kunming, Yunnan Province 650223 China; State Key Laboratory of Silkworm Genome Biology, Key Sericultural Laboratory of Agricultural Ministry, Institute of Sericulture and Systems Biology, Southwest University, 2 Tiansheng Road, Beibei District, Chongqing, 400715 China; Institute of Zoology, Chinese Academy of Sciences, 69 East Beichen Road, Chaoyang District, Beijing, 100101 China; Shenyang Agricultural University, 120 Dongling Road, Shenhe District, Shenyang Province, 110866 China; Center for Epigenetics, Johns Hopkins University School of Medicine, Baltimore, Maryland 21205 USA

**Keywords:** Silkmoths, Comparative transcriptomics, Silk proteins, Silk coloration

## Abstract

**Background:**

Silk has numerous unique properties that make it a staple of textile manufacturing for several thousand years. However, wider applications of silk in modern have been stalled due to limitations of traditional silk produced by *Bombyx mori.* While silk is commonly produced by *B. mori,* several wild non-mulberry silkmoths--especially members of family Saturniidae--produce silk with superior properties that may be useful for wider applications. Further utilization of such silks is hampered by the non-domestication status or limited culturing population of wild silkworms. To date there is insufficient basic genomic or transcriptomic data on these organisms or their silk production.

**Results:**

We sequenced and compared the transcriptomes of silk glands of six Saturniidae wild silkmoth species through next-generation sequencing technology, identifying 37758 ~ 51734 silkmoth unigenes, at least 36.3% of which are annotated with an e-value less than 10^−5^. Sequence analyses of these unigenes identified a batch of genes specific to Saturniidae that are enriched in growth and development. Analyses of silk proteins including fibroin and sericin indicate intra-genus conservation and inter-genus diversification of silk protein features among the wild silkmoths, e.g., isoelectric points, hydrophilicity profile and amino acid composition in motifs of silk H-fibroin. Interestingly, we identified *p25* in two of the silkmoths, which were previously predicted to be absent in Saturniidae. There are rapid evolutionary changes in sericin proteins, which might account for the highly heterogeneity of sericin in Saturniidae silkmoths. Within the six sikmoths, both colored-cocoon silkmoth specific transcripts and differentially expressed genes between the colored-cocoon and non-colored-cocoon silkmoths are significantly enriched in catalytic activity, especially transferase activity, suggesting potentially viable targets for future gene mining or genetic manipulation.

**Conclusions:**

Our results characterize novel and potentially valuable gene resources of saturniid silkmoths that may facilitate future genetic improvement and modification of mulberry silkworms. Our results suggest that the disparate features of silk--coloration, retention, strength, etc. --are likely not only due to silk proteins, but also to the environment of silk assembly, and more specifically, that stable silk coloration exhibited by some Saturniidae silkmoths may be attributable to active catalytic progress in pigmentation.

**Electronic supplementary material:**

The online version of this article (doi:10.1186/s12864-015-1420-9) contains supplementary material, which is available to authorized users.

## Background

Functional genomics has yielded an abundance of data on numerous plant and animal species while also providing novel techniques for isolating valuable traits or genes from these organisms. Further application of these techniques to economically valuable species holds the potential to vastly improve the quality of their produce by offering directions into future gene mining, genetic manipulation or breeding efforts. For example, silk produced by silkworms has long held historical, economic and cultural significance worldwide, especially in China where the mulberry silkworm *Bombyx mori* (*B. mori*) was domesticated nearly 5000 years ago. Today, China remains the world’s largest producer and exporter of *B. mori* cocoons and raw silks, which are predominately used textile manufacturing.

Advances in genomics and material sciences have also suggested potential uses of silk in medicine and security biomaterials, but several hurdles remain to these non-traditional applications, not the least of which being that the silk commonly produced from *B. mori* is less than ideal for such novel purposes or even more advanced silk textile production. For instance, *B. mori* silk has weak color retention, making it difficult to satisfy consumer’s textile demands; while outside of textiles utilization of this silk cannot meet tensile strength well for industrial application or use as a biomaterial. However, silk produced from other organisms often possess one or more of superior characteristics not present in *B. mori* silk, but these organisms’ silk production is poorly understood due to lack of genomic and genetic data. Aside from *B. mori*, several wild non-mulberry silkmoths, especially members of family Saturniidae, produce silks with unique features that are well suited to novel applications both within and outside of textiles. The family Saturniidae—the largest and arguably most spectacular in Lepidoptera—is comprised of over 1,500 different species [1], including economically important silk-producing moths such as Chinese and Japanese oak moth (*Antheraea pernyi* and *A. yamamai*), Assam silkmoth (*A. assama*) and Eri silkmoth (*Samia Cynthia ricini*). Of these, the yield of *A. pernyi* silk is next to that of *B. mori,* while the silk of *A. yamamai* and *A. assama* remains among the most valuable and expensive, being only used in top-end textiles due to its superior natural colors. Silk from *S. cynthia* is widely used in conjunction with cotton, hemp, wool or chemical fiber to create blended fabric. Likewise, other silk-producers such as *Actias selene* (*Ac. selene*) and *Rhodinia newara* possess unique characteristics with economic potential.

Structurally, silk glands of Saturniidae species are morphologically distinctive from those of *B. mori*. The former glands have relatively uniform curved morphology with no obvious distinction between the middle and posterior regions of the silk-glands, whereas the latter show drastic differences between the two regions, with the mid-silk glands swelling and straight and the posterior silk glands being curved [2]. Likely due to the morphological differences as well as differences in genetics and the underlying molecular structure of silk producing glands, silk produced by these Saturniidae species generally exhibits unique properties in terms of color, luster, strength, biological compatibility and cell adhesiveness, which either alone or in tandem make them commercially attractive for certain existing uses (i.e., textiles) or novel applications in medical applications [3,4]. In particular, silk from *Antheraea* moths such as *A. pernyi*, *A. yamamai* and *A. assama*, as well as *Ac. selene* and *R. fugax*, all share a markedly better tenacity, tensile strength and general toughness as compared to *B. mori. R. newara*, another species in the genus *Rhodinia*, has the nearly same cocoon features as those from of *R. fugax.* While the silk of *S. cynthia* has a weak tensile strength (making it difficult to spin), this lack of adhesion and strength make it suited for blending and creating artificial fabrics or materials. Similarly, some of these species’ silk also possess superior natural coloring and color retention that is significantly different from *B. mori*; for example, the green silk from *A. yamamai*, *R. fugax* [5], *R. newara*, and the glossy golden *A. assama* silk shows stable color retention during processing, making it invaluable for producing natural colored silks that do not require further artificial coloring or added processing costs. Despite myriad inherent advantages of the silk form these wild non-mulberry silkmoths, utilization of the silk is restricted by their non-domestication status or limited culturing populations.

Functional genomics has made it possible to begin investigating and exploiting genetic resources from the wild silkmoths and genetically modifying *B. mori* via gene mining or manipulation, with the promise of using domesticated species to produce silk with different properties usually associated with their wild counterparts [6]. However, to date, so far only low-coverage EST data are available for *A. assama*, *S. cynthia* and *A. mylitta* [7,8], and most studies on silk protein structure have only provided limited overviews [7,9-12]. The functional complexity of the silkmoth transcriptome for the Saturniidae silkmoths has not yet been sufficiently clarified. Recent applications of RNA-seq technology to eukaryotic transcriptomes have revealed an increasing number of novel transcripts and sequence variations [13,14], and also been used to analyze important traits-related gene pathways in organisms, even those without a presently available reference genome [15].

In the present study, we extended these efforts by generating a massive RNA-seq datasets of silk glands for six Saturniidae silkmoths, including *A. pernyi*, *A. yamamai, A. assama, S. cynthia*, *Ac. selene* and *R. newara.* Using these data, we conducted extensive comparative transcriptomics of these species’ silk glands and the mulberry silkworm *B. mori* to better characterize the genetic bases underlying the observed differences in the properties of silk produced by these species. Our results provide the basic data necessary for further explorations and potential uses of these species and the application of their silk.

## Results and discussion

### Sequencing and *de novo* transcriptome assembly

**Figure 1:**
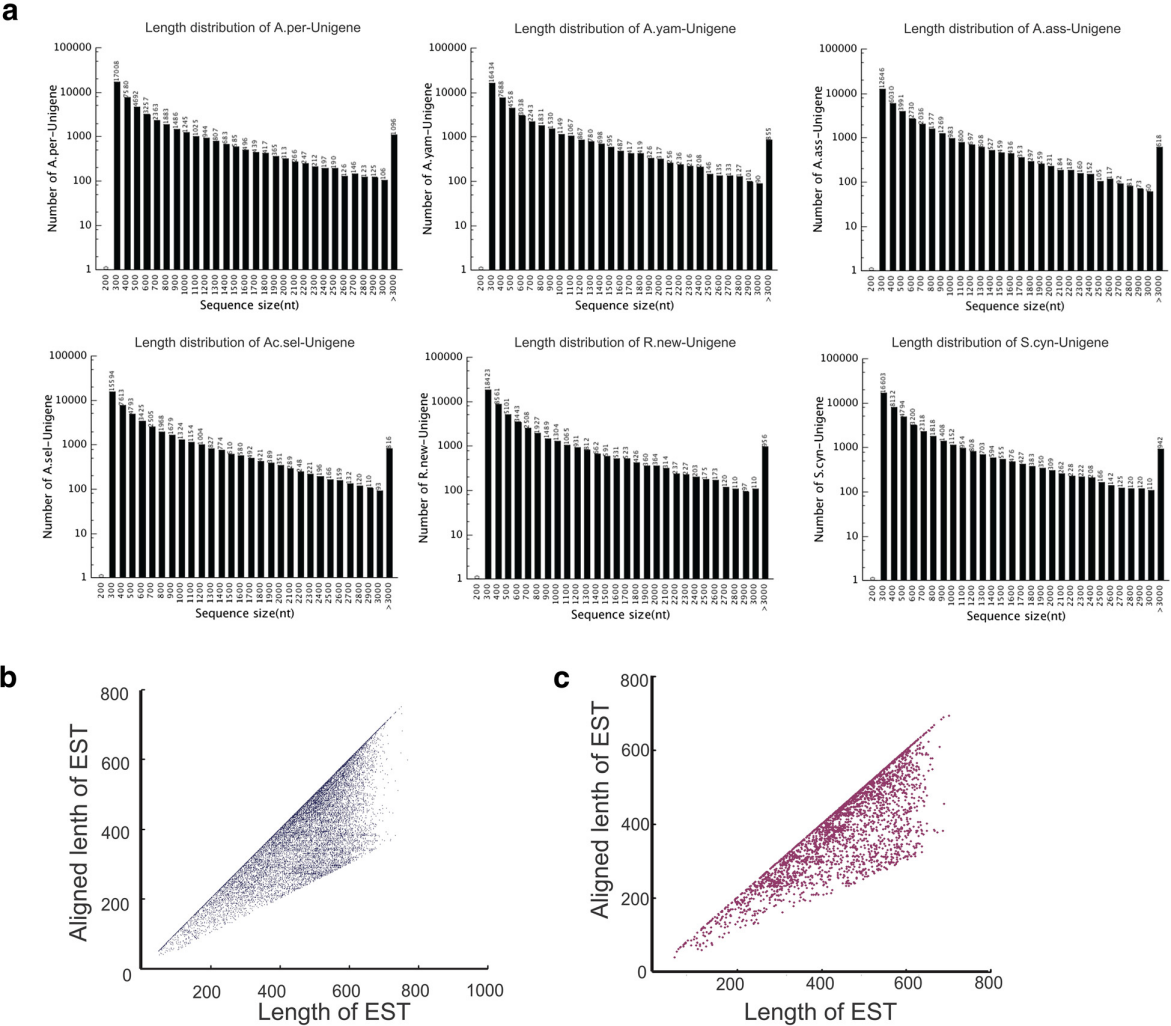
**Summary of transcriptome sequences. a**. Length distribution of the transcripts of six silkmoths. **b**,**c**. Comparison of *Antheraea assama* EST data from ten tissues **(b)** and silkgland **(c)** with *de novo* transcriptome. The aligned length vs total length of each EST hit in total tissues **(b)** and silk gland **(c)** were plotted, respectively. (Abbreviations: A.ass, *Antheraea assama;* A.per, *A. pernyi;* A.yam, *A. yamamai;* Ac.sel, *Actias selene;* R.new, *Rhodinia newara;* S.cyn, *S.* c*ynthia.* All following figures use these same abbreviations).

RNA-seq for silk glands from each 5-instar larva of the 6 silkmoths was generated on an Illumina HiSeq 2000 sequencer. The conducted 90 bp pair-end reads yielded 5.95 ~ 9.49 Gb of effective data for these samples (Table 1). Given the difficulties of transcriptome *de novo* assembly without reference genome information, we concurrently employed three *de Bruijn* graph based software packages—SOAPdenovo [16], Trans-ABySS [17] and T-IDBA 1.2.5 [18] to assembly transcriptome contigs. The contigs assembled by each software were merged by TGICL [19] as to generate final non-redundant unigenes for each species. We ultimately obtained 37,758, 48,422, 46,948, 48,053, 51,734 and 47,632 unigenes for *A. assama*, *A. pernyi*, *A. yamamai*, *Ac. selene*, *R. newara*, *S. cynthia*, respectively, with average length ≥ 660 bp (Table 1). For each species, at least 6500 unigenes are >1000 bp and 15,000 > 500 bp in length (Figure 1a). To further evaluate the *de novo* assembled transcriptomes, we took *A. assama* as an example, comparing the reported EST sequences (Genbank Accession number: FE952359-FG226965) with our assembled transcriptome unigenes. Totally, 35,191 ESTs from ten kinds of materials (i.e., embryo, brain, compound eye, epidermis, fat body, middle silk gland, posterior silk gland, midgut, ovary and testis from larvae) were blasted against our assembled contigs with blastn, and 18,978 (53.9%) of ESTs showed ≥ 90% identity with more than 50% matched length of the corresponding ESTs. Likewise 2735 of the 3043 ESTs (89.9%) from middle and posterior silk gland were represented by our assembly. We plotted the aligned length vs total length of each EST hits in total tissues (Figure 1b) and silk gland (Figure 1c), respectively. The results indicate that the majority of ESTs show fairly high aligned length. Moreover, we used the 248 core eukaryotic genes (CEG) [20] as a reference to evaluate the quality of the assemblies. The CEGs were well represented in the assembled transcriptomes of the *A. assama*, *A. pernyi*, *A. yamamai*, *Ac. selene*, *R. newara*, *S. cynthia*, with significant matches (alignment length ≥50% CEG length & e-value <1 × 10^−20^) to 85.85%, 84.21%, 89.19%, 89.85%, 88.72% and 81.48% of the CEGs, respectively. Taken together, high consistency between ESTs and *de novo* assembled transcriptome and well-characterized representations of CEGs suggests that our multi-assembly approach could be used to construct transcriptomes with a reasonable completeness and quality by using deep RNA-seq data.

**Table 1:** **Summary of the six silkmoth silk gland transcriptome**

	**A. per**	**A. yam**	**A. ass**	**Ac. sel**	**R. new**	**S. cyn**
Clean reads	77,694,700	104,865,480	66,063,176	102,577,780	105,422,226	82,495,146
Effective data (bp)	6,992,523,000	9,437,893,200	5,945,685,840	9,232,000,200	9,488,000,340	7,424,563,140
Total number of contigs (Unigenes) (bp)	48,422	46,948	37,758	48,053	51,743	47,632
N50 (bp)	1068	1011	950	1020	995	1000
Mean length of contigs (Unigenes) (bp)	691	669	660	686	661	668

### Transcriptome annotation

Firstly, we compared the unigene sequences of the six silkmoths with the predicted 14,623 protein-coding silkworm (*B. mori*) genes that were built by merging different gene datasets using GLEAN in the SilkDB [21], respectively. Totally, 97.55 ~ 98.21% of the assembled transcripts have matched homologous hits (e < 0.00001) with 74.68 ~ 82.38% of the silkworm genes (Additional file 1: Table S1). Complementing the data, we also used the published silkworm’s transcriptome data unmapped to the silkworm genome [14] to carry out the same comparison and found that 21.38 ~ 23.75% of the assembled transcripts of the silkmoths have matched homologous hits (e < 0.00001) with 27.55% ~ 29.30% of the unmapped silkworm transcripts. Overall, these comparisons also suggest a high-quality assembly. Secondly, unigenes of the six silkmoth transcriptomes were searched with BlastX against NCBI non-redundant protein database (nr), Swiss-Prot, KEGG and COG consecutively, with the similarity cutoff e value < 0.00001. Totally, 36.3% ~ 42.0% unigenes have matched homologous hits. Unigenes with no significant hit were further scanned by ESTscan for coding sequence (CDS) prediction and 2630 ~ 3040 of these unigenes had ≥ 200 bp coding region (Table 2), in which the N50 of CDS reached 1 kb, and more than 200 unigenes had a CDS length over 3 kb.

**Table 2:** **Summary of annotation results of the six silkmoth annotated unigenes**

	**A. per**	**A. yam**	**A. ass**	**Ac. sel**	**R. new**	**S. cyn**
All	17583 (36.3%)	19178 (40.8%)	15854 (42.0%)	18347 (38.2%)	19013 (36.7%)	17783 (37.3%)
Nr	17498	18418	15829	18361	18907	17778
SwissProt	13659	15367	12612	14290	14423	13765
GO	4297	6101	5284	5077	6023	5424
KEEG	11077	11752	10140	11610	11645	11081
COG	6312	7320	5739	6557	6629	6426
With predicted CDS (> = 200 bp)	2630	3040	2382	18347	2688	2436

The comparatively highly complete transcriptomes of the six silkmoths provide valuable resources for further understanding the genome of non-mulberry silkmoths in Saturniidae. Totally, our methods annotated at least 4297 unigenes in GO and 5739 in COG categories (Table 2) based on sequence homologies. In three main categories (biological process, cellular component and molecular function) of the GO classification, “metabolic process and cellular process”, “cell and cell part” and “binding and catalytic activity” were dominant in silk glands (Additional file 2: Figure S1). Among the 25 COG categories, “general function prediction” represented the largest group, followed by “translation, ribosomal structure and biogenesis”, “replication, recombination and repair” and “transcription” (Additional file 2: Figure S2). The predominance of such GOs and COGs suggest that the silk glands of 5th instar of these silkmoths are highly active in both protein and nucleic acid metabolism, which are necessary for protein synthesis.

### Diversity of transcribed gene sequences

**Figure 2:**
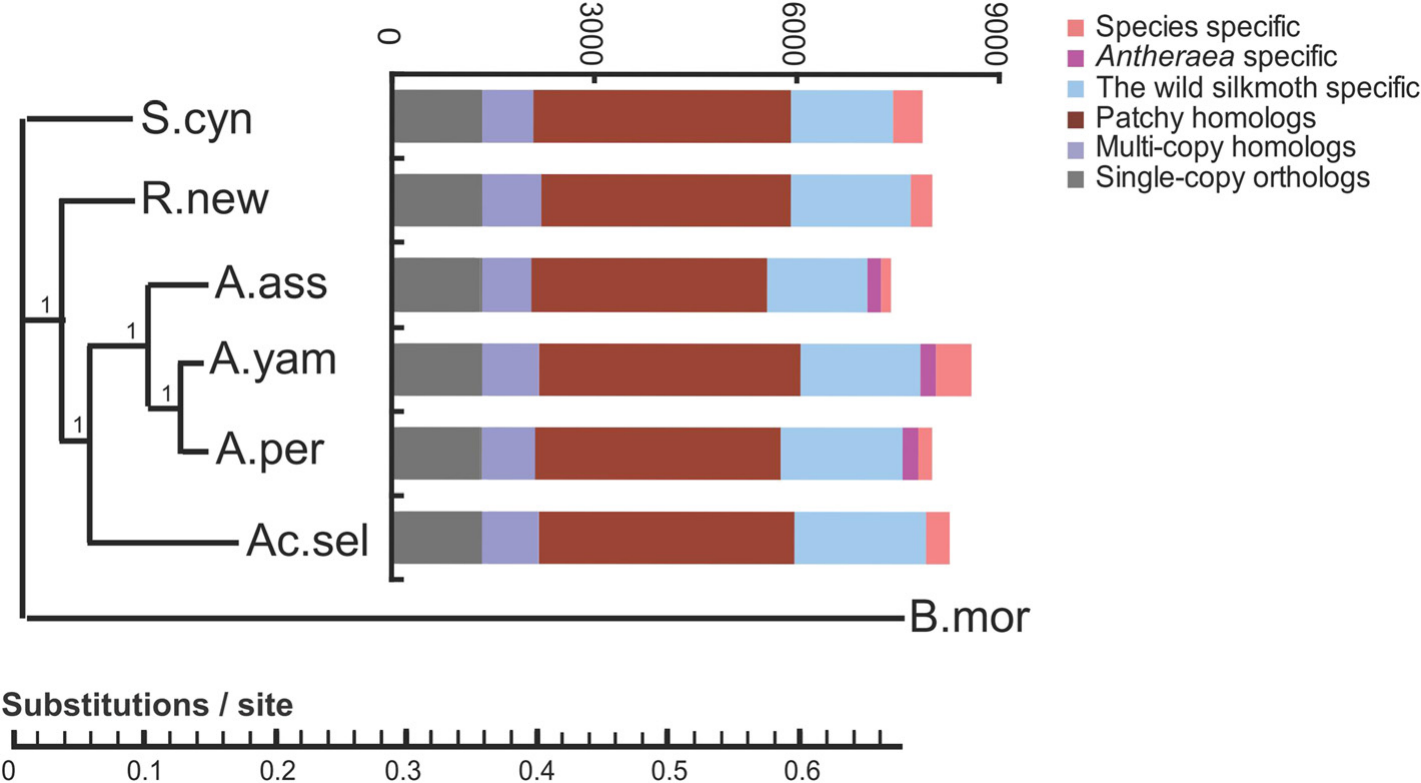
**Transcriptome of the six silkmoths.** Phylogenetic tree based on Bayesian inference analyses of a concatenated alignment of single-copy genes (left) and homology relationships in the six silkmoths (right), using *B. mori* as outgroup. Bayesian posterior probability was shown for each node.

We used TreeFam-4.0 [22] to define a gene family as a group of genes that descended from a single gene in the last common ancestor of a considered species, or more succinctly, of genes belonging to one homologous group. In total, 17,357 gene families were identified among the six analyzed silkmoths and *B. mori* (Additional file 3: Data S1). Among the annotated genes, 30.1% ~37.2% were homologs conserved in all the six silkmoths and *B. mori* (Figure 2), including 1342 single-copy orthologs (1:1:1:1:1:1:1 orthologs), 737 ~ 858 multiple-copy homologs and 3476 ~ 3866 patchy homologs (single-copy orthologs in at least one species and multiple-copy homologs in at least one other species). We found 929 gene families (1428 ~ 1797 homologs) specific to Saturniidae silkmoths, 181 (216 ~ 236) specific to the genus *Antheraea*, and a further 65 ~ 207 (159 ~ 513) that are species-specific (Figure 2). Saturniidae specific genes were enriched in binding and enzyme regulator activities, and primarily involved in growth progress (Additional file 2: Figure S3), likely reflecting the distinctive silk gland development of the Saturniidae silkmoths and consequent morphological differences observed between them and *B. mori.* These Saturniidae specific genes resources are potential targets, not only for further exploration on genetic bases of silk gland differentiation but also for potential genetic modification on *B. mori.*

Based on the most conserved single-copy orthologs, we reconstructed the phylogenic tree of the six silkmoths, via Bayesian inference method (Figure 2, left), Maximum likelihood method (Additional file 2: Figure S4a) and Neighbor-Joining (NJ) method (Additional file 2: Figure S4b), respectively. The three trees show highly similar topologies. The trees indicated that *Antheraea* genus clustered together, and as expected, that *S. cynthia* was most distantly related to the other silkmoths. Within *Antheraea*, *A. pernyi* and *A. yamamai* were closer to one another than to *A. assama*. A previous report suggested that the former two species could be crossed to produce F1 progeny [23], and other studies found that their silk fibroins are highly similar, underscoring the strong relationship between the two species [9,10]. The trees also showed that *Ac. selene* is next closest to *Antheraea spp*. Overall, the phylogenetic tree was generally consistent with previous studies using single gene or mitochondrion sequences as molecular markers [24,25], but provided more information, making it a more solid phylogenetic analysis based on large-scale transcriptome sequences.

Although the final tree is not a fully resolved tree, with a polytomy in the root node corresponding to the *B. mori* linkage and the Saturniidae linkage (Figure 2, left), this uncertainty from the tree could be easily clarified in follow-up studies since the phylogenetic relationship between the two taxa is fairly clear.

### Silk protein genes

Silk proteins produced by the silkworm *B. mori* include fibroin consisting of a heavy chain (H-fibroin) and a light chain (L-fibroin), sericin, and a glycoprotein P25 known as fibrohexamerin. While the structure and composition of silk filament proteins (fibroin and P25) may well be ancient and conserved in Lepidoptera [26], it was thought that an extreme variation of the silk structure occurs in the Saturniidae silkmoths, as they possess modified H-fibroin and lack L-fibroin and P25 [26].

**Figure 3:**
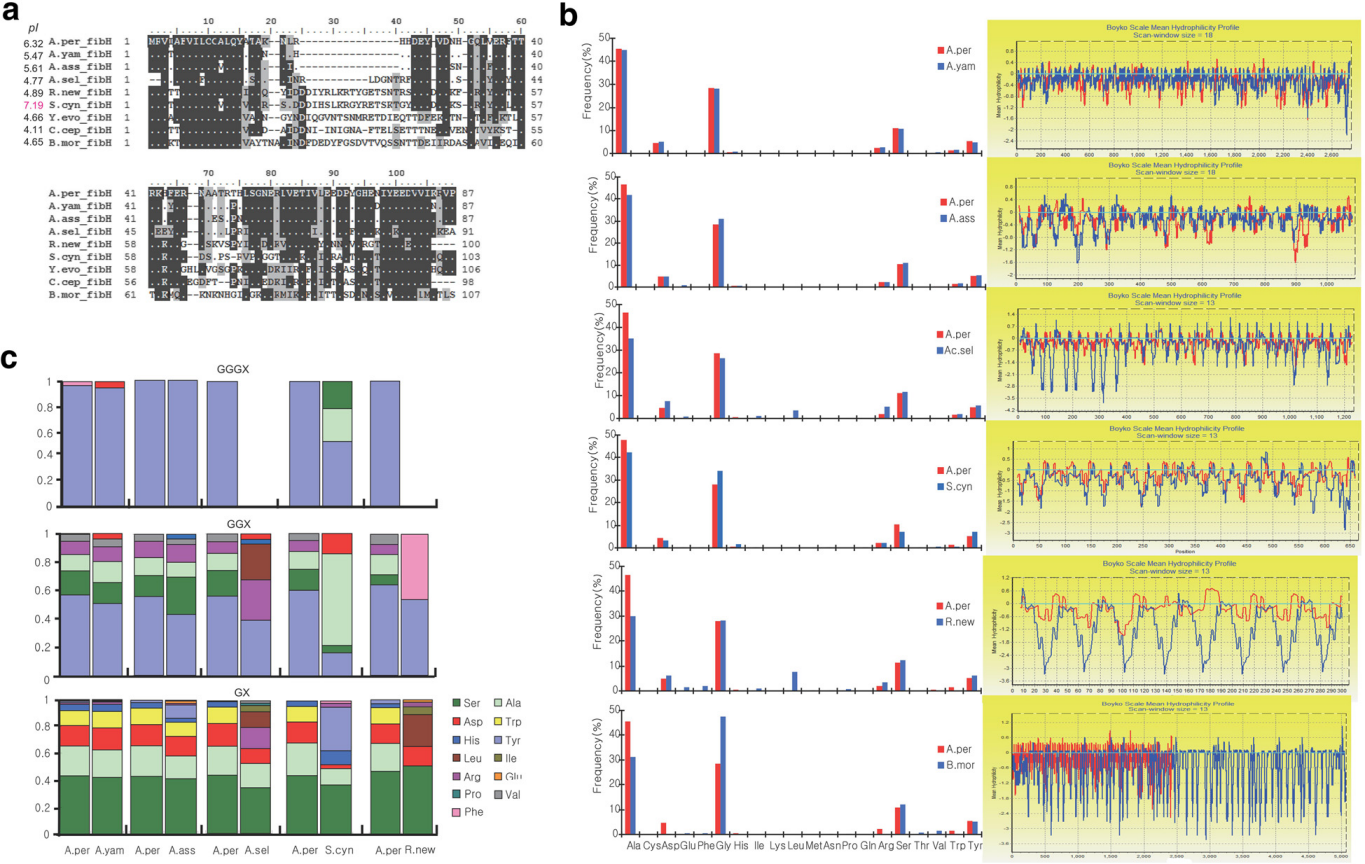
**Characteristics of Fibroin H protein. a**. Deduced amino acid sequences of N-terminal. Isolectric points are indicated ahead of each gene name. **b**. Amino acid composition (left) and hydrophilicity profile of repeats region. Repetitive region of *A. pernyi* as a reference and to generate pair-wise comparison on aligned sequences and only aligned regions were analyzed. Blue profiles showed the hydrophilicity profiles of aligned regions of *A. pernyi* and the red ones showed the hydrophilicity profiles of aligned regions in the other species. **c**. Amino acid composition of X (X refers to amino acids other than G) in repetitive GGGX quadruplexs, GGX triplets, GX diplets in the repeat region. (Abbreviations: Y.evo, *Yponomeuta evonymellus*; C.cep *Corcyra cephalonica*).

Due to the highly repetitive nature of H-fibroin protein sequence, we were unable to obtain the whole sequences of the H-fibroin transcripts for the six studied silkmoths. However, we were able to obtain and compare reliable non-repetitive N terminus (87 ~ 103 amino acid) and the partial repetitive regions. Molecular evolution of the N terminus was analyzed by PAML 4.7 [27], using the branch model. The N terminus of silkworm *B. mori* (AF226688.1) and other two Lepidoptera species (ACX50394.1 for *Corcyra cephalonica* and BAE97695.1 for *Yponomeuta evonymellus*) were also included for further analyses. The average ratio of nonsynonymous substitutions to synonymous substitutions (dN/dS, ω) of the N terminus on all branches was 0.29 under the one ratio model, supporting the hypothetical functional constraints of N termini across the phylogeny. The likelihood ratio test to compare the fitness of free ratio model and one ratio model did not show significant fitness of the former (2Δ*l =* 14.88, p > 0.5), suggesting an overall similar dN/dS of the N terminus on all branches. The first 14 residues in the N terminus, which is the first exon in *A. pernyi* [9], are quite conserved between all the six Saturniidae species and the Bombycidae *B. mori* (Figure 3a). This conservation may conceivably occur quite widely across silk producing Lepidoptera species (Figure 3a) [26], suggesting its important functional significance.

Our results further showed variations in the remaining amino acids in N termini among the different species. For instance, we observed a relatively uniform deletion in the three *Antheraea* species (Figure 3a). In the other species, sequences in this region are diverse, and their functional implications need further exploration (Figure 3a). We calculated isoelectric points (pI) of the N termini of the six studied silkmoths and *B. mori* as well as the other two Lepidoptera species, *Corcyra cephalonica* and *Yponomeuta evonymellus* and found that pIs of the silkworm *B. mori* and the other two Lepidoptera species are similar (~4.4), while there were great variations on pIs for the six Saturniidae species, ranging from 4.77 (*Ac. selene*) to 7.19 (*S. cynthia*) (Figure 3a). The N terminus of silk H-fibroin was previously suggested to play important roles in mediating the assembly of silk in response to a decrease in pH [28]; accordingly, the diversification among the Saturniidae silkmoths suggest a diversification in silk assembly mechanism(s).

Based on pI of N terminus, it is intriguing to consider that silk assembly in *S. cynthia* seems to be in a slightly basic rather than the more common acid environment seen in most lepidoptera species, suggesting a possibly different silk assembly mechanism in *S. cynthia* that may influence the unique properties of its silk. Given that the silk of *S. cynthia* is comparatively weak and difficult to spin, the difference in composition and structure of N terminus may well imply that the silk features may, in part, be due to the relative acidity of the production environment. This possibility, that environmental acidity may influence the nature of the silk produced within it, may have profound implications for future investigations into silk textile production and novel applications. Further targeted investigations within and outside of genomics may explain this more clearly, especially since the repetitive motifs following the N terminus are traditionally understood to be the determining factor of silk fiber properties [9,26,29,30].

In accordance with previous studies [5,9,10], our results indicate that the all six Saturniidae silkmoths have poly-alanine repetitive motifs (Additional file 4: Data S2) and not poly-glycine-alanine repeats in *B. mori,* similar to dragline protein of spiders [31]. These poly-alanine repeats are more hydrophobic than poly-glycine-alanine of *B. mori,* likely because poly-alanine β-sheets impart a higher binding energy than poly- glycine-alanine β-sheets, thereby potentially contributing to the better tenacity [32]. However, the uniform poly-alanine repeat cannot explain the differences in silk properties between all six of the studied silkmoths. To investigate this topic further, we generated pair-wise comparison on aligned sequences of the repeated regions, using the repeated region of *A. pernyi* as a reference. We found that the amino acid composition is similar among the three *Antheraea* silkmoths. However, in *Ac. selene* and *R. newara*, alanine is comparatively less frequent while leucine is remarkably frequent (Figure 3b). Additionally, *Ac. selene* fibroin repeats contain more arginine and aspartic acid (Figure 3b). Consistently, the hydrophilicity profiles of the fibroin repeats were similar among three *Antheraea* silkmoths but drastically different between *R. newara* and *A. pernyi*, *Ac. selene* and *A. pernyi*, respectively*.* Specially, *R. newara* fibroin repeats are much more hydrophilic than *A. pernyi* and even somehow similar to those of *B. mori*; *Ac. selene* fibroin repeats in upstream region, are more hydrophilic whereas as to those in the downstream region, shift of hydrophilicity between the hydrophilic and hydrophobic blocks are more intense than that of *A. pernyi* (Figure 3b).

Since hydrophilicity of silk protein is important for stability of the silk dope [33], these differences in hydrophilicity suggest that silk from *R. newara* and *Ac. selene* have different properties compared with the *Antheraea* silkmoths (Figure 3b). We further noticed that the amino acid composition of the non-poly-alanine blocks of the six silkmoths are predominantly composed of hydrophilic amino acids with many repetitive GGGX (X refers to amino acids other than G) quadruplexes, GGX triplets, GX diplets; as expected, amino acid composition of these quadruplexes, triplets or diplets was similar among the three *Antheraea* silkmoths but diverse among the other three silkmoths from the three different genera (Figure 3c). The peptide motif GGX was previously hypothesized to conform to a 3_1_-helix that is capable of forming an interhelix hydrogen bond, which may help to explain the mechanical properties of the silk [34]. Functional significances of the other two peptide motifs are still unknown. However, at this juncture, the observed intra-genera similarities and inter-genera differences on amino acid composition of these peptide motifs may provide cues for better understanding and characterizing the silk properties of the six silkmoths we investigated in this study.

**Figure 4:**
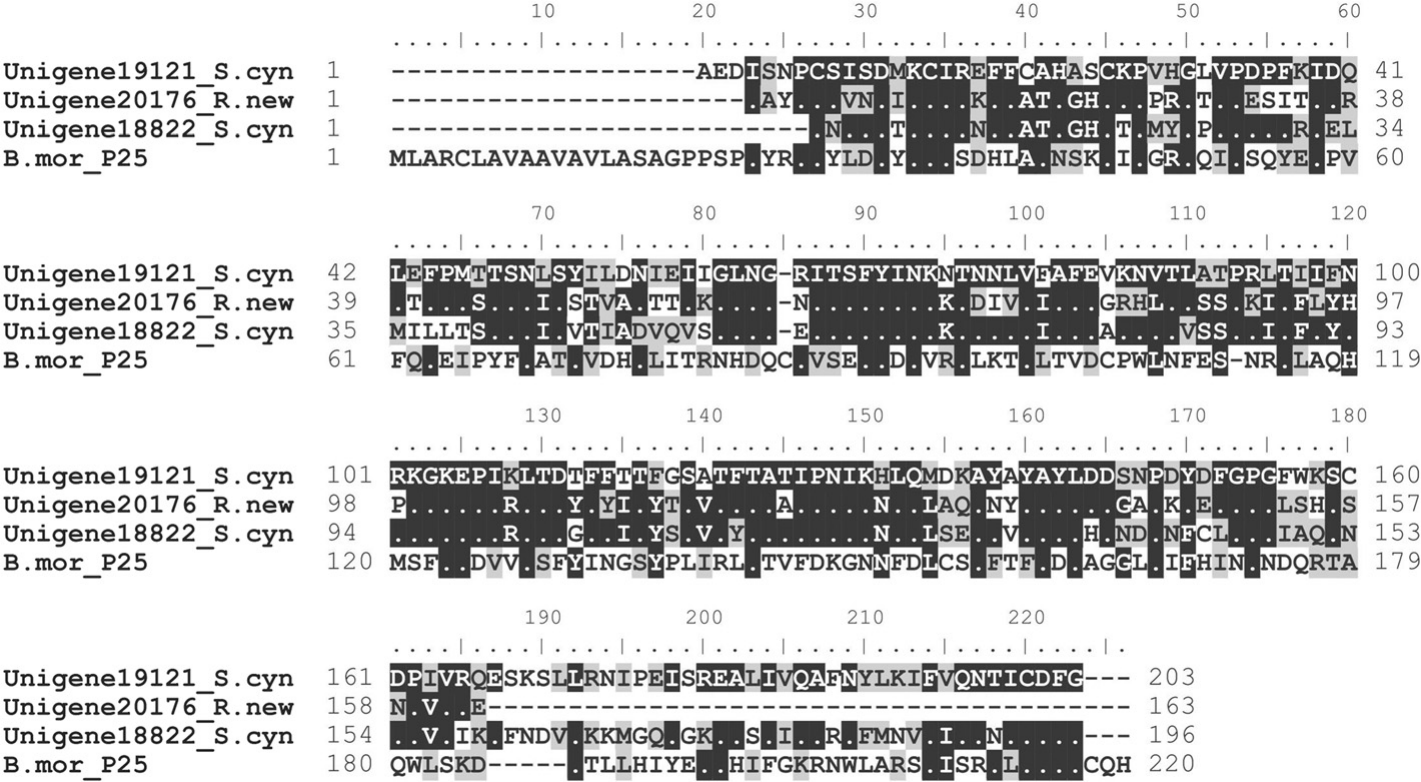
**Deduced amino acid sequences of P25.**

Interestingly, we identified P25 (Figure 4) homologs in two silkmoths (*R .newara* and *S. cynthia*), though Saturniidae silkmoths were previously suggested to lack this protein [26]. We noticed that expression of P25 homologs were low in *R. newara* (RPKM = 3.31) but rather high in *S. cynthia* (RPKM = 66 for one transcript and 317.2 for the other), suggesting there could be variation in silk composition and further silk structure within Saturniidae silkmoths. In the silkworm *B. mori*, P25 links H-fibroin and L-fibroin through disulfide bond to form Cys-linked heterodimers in *B. mori* [35]. As expected, we did not identify L-fibroin in any of these silkmoths, consistent to previous reports [26], leaving the functions of P25 in these two silkmoths enigmatic, especially since we noticed the P25 homologs were highly expressed in *S. cynthia*. Potentially, P25 may contribute to the possible consequent novel structure of the *S. cynthia* silk, but as our supposition on the acidity of the this species’ silk producing environment, more investigations are needed to explore which of these differences is potentially responsible for the traits inherent in *S. cynthia* silk.

**Figure 5:**
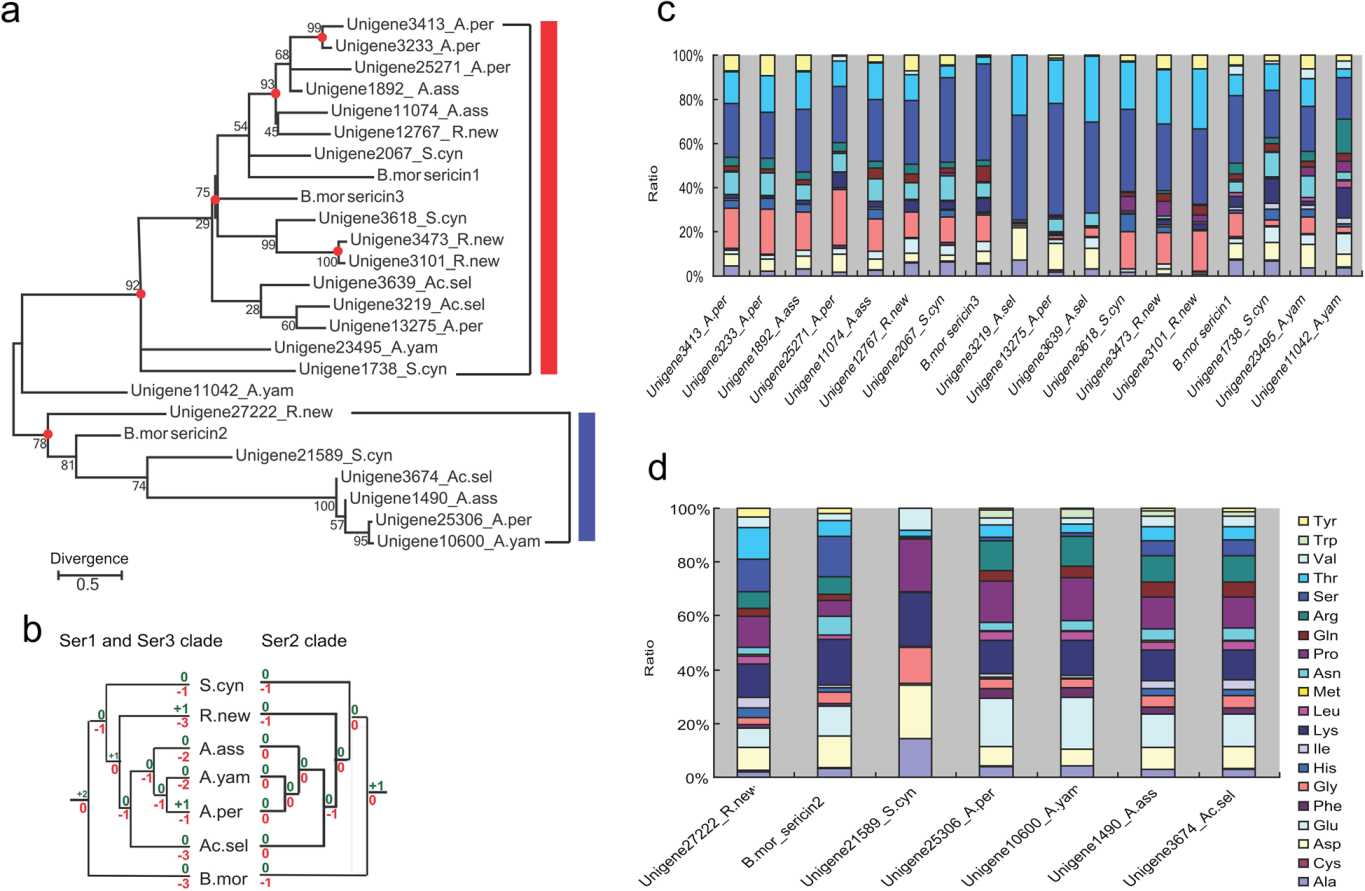
**Characteristics of sericins. a**. Phylogenetic tree of sericin homologs based on maximum likelihood method with bootstrap replicates set as 1000. Numbers near branches indicate the bootstrap values for the given clades. The red dots at internal nodes denote where gene duplication events have occurred, supported with bootstrap value >75. **b**. Evolutionary events of the sericin genes. Numbers of gene duplication (shown in green after “+”) and loss (shown in red after “-”) events were inferred for each internal node as well as for the seven species. Red box represents Ser-1 and Ser-3 homologs and blue box denote Ser-2 homologs. **c**. Amino acid composition of Ser-1 and Ser-3 homologs. **d**. Amino acid composition of Ser-2 homologs.

Another factor previously postulated to influence silk was the presence of sericin proteins [36], especially, in some Saturniidae species. These species’ cocoons have a unique structure - peduncle, wherein sericins are thought to increase the strength of silk [37]. Unfortunately, the role or presence of sericin in Saturniidae silkmoths is still poorly characterized, without any sericin gene identified to date. In silkworms, sericins are a family of cocoon proteins coded by two sericin genes, *Ser-1* [38] and *Ser-3* [39]. Another sericin gene, *Ser-2* was previously suggested to encode the major coating proteins of non-cocoon silk, which is highly adhesive and spun during the larval stage [40,41]. Here, we used the three *Bombyx* sericin proteins as references and successfully identified homologs in the six silkmoths via BLASTP (Additional file 5: Data S3). Further phylogenetic analysis indicated a clear Ser-2 cluster, which includes homologs from all the six silkmoths. However, our analysis did not conclusively distinguish Ser-1 and Ser-3 homologs, potentially because of the relatively high similarity between these two genes and/or high inter-specific divergence of the homologs (Figure 5a). To better understand the evolutionary events of sericin genes in the silkworm *B. mori* and the six silkmoths, we reconciled the obtained Maximum likelihood tree with species tree and detected a dynamic evolutionary history of these proteins featuring obvious gene birth and death (Figure 5b). As for Ser-2 proteins, it seems that an ancient duplication event occurred in the root node and then gene loss occurred in the early diverged species. For Ser-1 and Ser-3 proteins, a comparatively more rapid duplication/loss event(s) seems to have occurred, with two duplication events at the root node and subsequent gene loss events in nearly every internal node and species. Duplications also occurred at the one internal node and two species linkages. The rapid evolutionary changes in sericin proteins may potentially account for the high heterogeneity of sericin in Saturniidae silkmoths [4,37].

Generally, the Ser-1 and Ser-3 homologs in the six silkmoths were serine rich (Figure 5c), similar to *B. mori* Ser1 and Ser3 proteins. We also found patterns among the Ser-2 proteins in the six studied silkmoths that are similar to those of *B. mori*, with the ratio of serine being relatively low, and highly positive charged lysine relatively high. Similarly, the proteins also contain many negatively charged amino acids, such as aspartic acid and/or glutamic acid (Figure 5d). High occurrences of both positively and negatively charged residues help to allow for electrostatic interactions with molecules in the substrate surfaces, which may account for the adhesiveness of Ser2 protein [40]. We also observed high ratios of proline (Figure 5d). Previous studies found that high incidence of proline is capable of stabilizing bends in the peptide chain and thereby hinder interactions with residues of the opposite charge [41]. Conceivably, these incidences may play a contributing role in this protein’s adhesiveness. Despite a general conservation, we noticed that there were variations of amino acid composition in some homologs. For example, Ser-1/Ser-3 homologs in *Ac. selene* and *A. pernyi* have no or very few glycines; Ser 2 homologs in *S. Cynthia* had no glutamic acid. These differences in amino acid composition seem to reflect the diversification of the sericin proteins in the Saturniidae silkmoths and as posited previously [36,37], may play roles in the divergence of their silk features. The gene resources provided here will likely facilitate further gene mining and application of these silk proteins to genetic engineering of silkworms.

### Genes and potential mechanisms related to silkmoth cocoon coloration

Alongside strength and structure of the various silkmoths silks, superior coloration and color retention of A. *yamamai, R. newara* and *A. assama* silk makes these species particularly advantageous in textile manufacturing. Conversely, the cocoons of *A. pernyi* and *Ac. selene* appear dark yellow, but this coloration does not remain stable during processing. Why the earlier mentioned three species maintain a stable coloring but others do not is unclear yet. We suspect there may be special active pigmentation binding mechanisms in the silk fiber of A. *yamamai, R. newara* and *A. assama* that are quite different from those of *B. mori* or other silkmoths whose silk either does not possess any particular coloration or is not able to stably retain it. In *B. mori,* previous studies found two kinds of exogenous pigments absorbed from dietary mulberry leaves, namely carotenoids and green flavonoids [42-44]. Both of these are weakly bound and not directly bound to fibroin proteins, potentially causing the cocoons to be easily decolored during processing.

The existing studies on coloration in *B. mori* have found two genes controlling cocoon coloration, quercetin 5-O-glucosyltransferase encoded by *Green b* gene accounting for green cocoons, and carotenoid-binding protein (CBP) encoded by the *Yellow blood* gene that controls yellow cocoons [42,44]. However, the underlying genetic mechanism in Saturniidae silkmoth silk coloration are mostly unknown, except for *A. yamamai* and *R. fugax*, which both have an endogenous pigment known as blue bilin alongside the yellow carotenoids involved in cocoon coloration [45,46]. Previously, a blue pigment protein, named bilin-binding protein (BBP), was previously reported to play roles in bilin metabolism in some species of Lepidoptera [47,48]. To further test if these may be present in some form among our six selected species, we used Lepidoptera BBP sequences available in GenBank (BAM20268 & BAM19354) and *Bombyx* CBP (BAF56876) as references to search for related homologs. CBP appears to be relatively conserved, but BBP homologs are diverse among the Saturniidae silkmoths (Additional file 2: Figure S5a and S6a). Given that silk produced by A. *yamamai, R. newara* and *A. assama* is stably colored, we considered these three silkmoths as a colored cocoon group, and the other three silkmoths (*A. pernyi, Ac. selene,* and *S. cynthia*) as a non-colored group. In our analysis of these two groups, we did not observe any obvious differential expression of the homologs in colored cocoon species as compared with non-colored cocoon ones, either for *cbp* or for *bbp* (Additional file 2: Figure S5b and S6b). That said, curiously the *bbp* gene in *A. assama* was extremely highly expressed (Additional file 2: Figure S5b) while the *cbp* genes in *R. newara* were highly expressed as compared with those in other species (Additional file 2: Figure S6b). Though an interesting finding, our analysis was unable to clarify the functional impacts of such gene expressions. Further exploration of this phenomenon may be warranted, which may better elucidate the role of such mechanisms in silk coloration and color stability. Collectively, while these results are largely preliminary, they imply that pigment binding proteins are unlikely to solely account for stable silk coloration among the species in the colored cocoon group, though they may play some yet unknown role.

**Figure 6:**
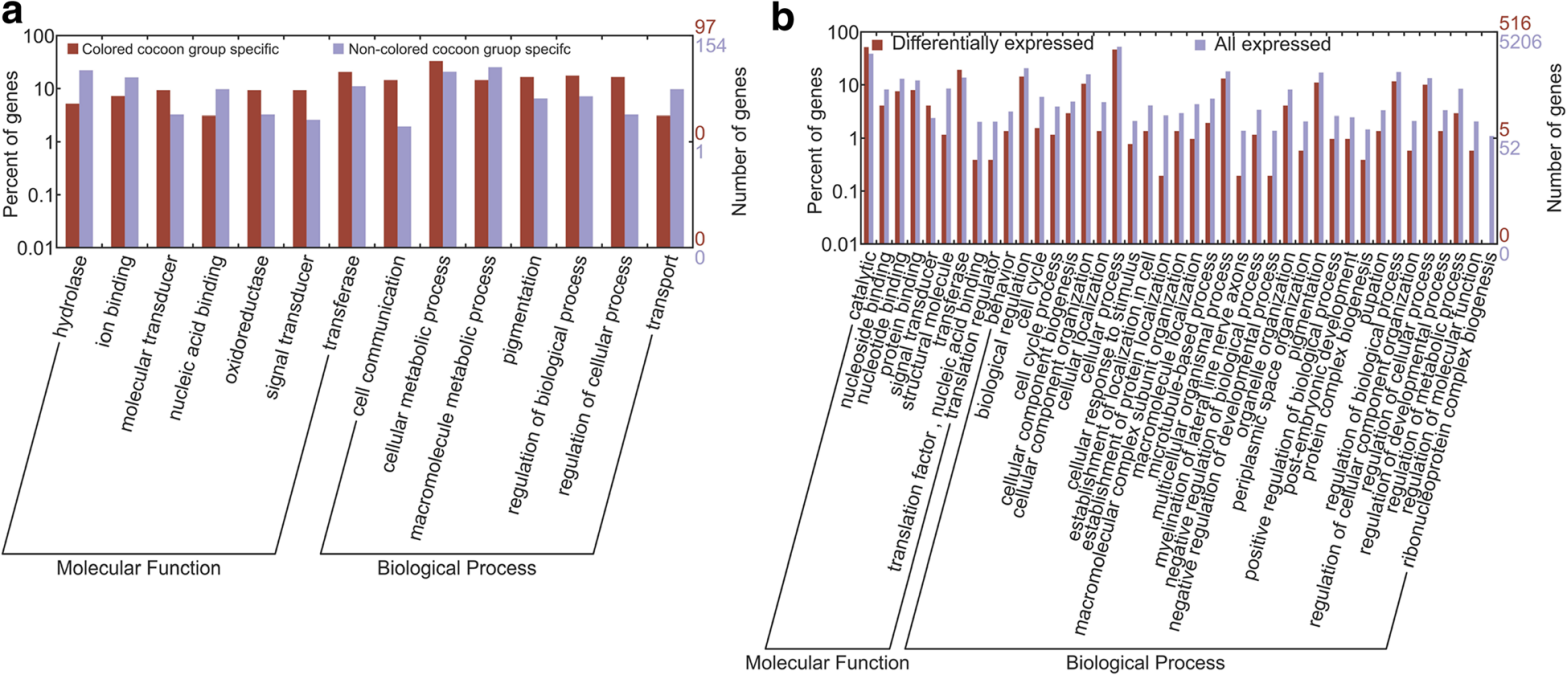
**Go enrichments of the colored-cocoon group specific (a) and colored- cocoon group differentially expressed genes (b).**

Further comparative transcriptomics between the colored and non-colored group of silkmoths detected 97 gene families specific to the colored-cocoon group and 136 to the non-colored-cocoon group, respectively. The relationship between the color retention and each of gene families identified were evaluated based on correlation analyses, using phylogenetically independent contrasts [49]. Totally two and five out of the 97 and 136 gene families didn’t show significant relationship with color retention, respectively, and were excluded from further GO enrichments analysis. Generally, as compared with non-colored-cocoon group specific genes, colored-cocoon group specific genes were significantly enriched in transferase, oxidoreductase and molecular transducer activity, and also enriched in such biological processes as pigmentation, cell communication, cellular metabolic process and regulation processes (Figure 6a). These results suggest these genes may be actively involved in enzymecatalysed reactions in silk gland and/or cocoon pigmentation. We further attempted to identify the genes involved in stable cocoon coloration by comparing expression level of the gene families between the two groups. Totally we identified 324 differential expressed gene families between the two groups, corresponding to 1614 genes in the colored-cocoon group. These genes are enriched signal transducer and catalytic activity, especially transferase (Figure 6b).

The consistent enrichment of genes with transferase activity in colored-cocoon specific and differentially expressed genes is quite intriguing. The observed result is somewhat supportive of our hypothesis of active pigmentation binding mechanisms in silk fibers, particularly in that it suggests that substantial catalytic reactions occur within the silk glands of colored-cocoon group but not (or at least less-so) within the non-colored-cocoon group. We suggest that stable coloration in silk could be attributed to active catalytic progress in pigmentation. Frankly, without comprehensive comparative transcriptomics on different non-silk tissues within a species, we are unable to exclude background expression to narrow down the potential candidate genes identified from each species. Meanwhile, without replicates for each species, we cannot distinguish fluctuation in the expression of individuals. Considering these limitations, the association between stable coloration and active catalytic progress in pigmentation is only conjecture. Paired alongside our other results, however, this finding may prove a viable target for further inquiry. Undoubtedly, future efforts on more comprehensive comparative transcriptomics and even comparative genomics among the silkmoths will shed significant light on the mechanism underlying cocoon coloration.

## Conclusion

In conclusion, the present study provided a substantial amount of transcriptome data and gene resources of the silk glands belonging to economically important wild silkmoths. Our annotation analysis indicated that the silk gland of 5th instar of these silkmoths are extremely active in protein and nucleic acid metabolism for protein synthesis, and that there are substantial genes specific to Saturniidae which are primarily involved in development processes, likely reflecting the distinctive silk gland development the Saturniidae silkmoths and consequent morphological differences observed between them and *B. mori.* Our study demonstrates intra-genus conservation and inter-genus diversification of silk protein features in these six silkmoths. From the isoelectric points analysis of N terminus of fibroin proteins, we suspected that the weak strength of *S. cynthia* silk is in part, due to the relatively acidity of the production environment, suggesting that silk with greater tensile strength potentially requires a more neutral environment for production. Our study also provides a suggestive genetic basis of active catalytic progress that may be associated with stable cocoon coloration. Collectively, these findings should prove useful in further comparative genomic explorations on the silk production, evolution of silk producers, and most especially further mining for genes involved in different aspects of silk-quality.

## Methods

### Sampling

Female silkmoths were collected by light trap. The fertilized individuals were kept at room temperature. Eggs laid were collected and disinfected. The eggs were hatched in incubator at 28°C. The larvae were fed with fresh leaves until the 5th instar. Silk glands from one 5th instar larva for each species were dissected for RNA extraction.

### RNA extraction and transcriptome sequencing by high throughput RNA-seq

Total RNA was isolated using TRIzol total RNA isolation system (Invitrogen) according to the manufacturer’s protocol. RNA integrity was confirmed using the 2100 Bioanalyzer (Agilent Technologies) with a minimum RNA integrated number value of 8. The samples for transcriptome analysis were prepared using Illumina kit following manufacturer’s recommendations. Briefly, mRNA was purified from 6 μg of total RNA using oligo (dT) magnetic beads. Following purification, the mRNA was fragmented into small pieces using divalent cations under elevated temperature and the cleaved RNA fragments at 200–250 bp were used for first strand cDNA synthesis using reverse transcriptase and random primers. This was followed by second strand cDNA synthesis using DNA polymerase I and RNaseH. cDNA fragments then went through an end repair process and ligation of adapters. The products were purified and enriched with PCR to create the final sequencing cDNA library. Both ends of library were sequenced on the Illumina sequencing platform HiSeq 2000.

### *De novo* transcriptome assembly

The raw reads were cleaned by removing adaptor sequences, duplicated reads and low quality reads. *De novo* assembly of the short reads was performed using SOAPdenovo, Trans-ABySS and T-IDBA, respectively. As to SOAPdenovo, K-mers 19, 21 and 23 were tested and k-mer 23 was used at last because of its best performance. Other parameters were set as default. The longest assembled sequences containing no Ns are considered as contigs. As to Trans-ABySS, we first used ABySS to generate transcriptome assemblies using odd k-mer values ranging from 23 to 57. Then we used Trans-ABySS to merge those multi-k-mer assemblies to contigs with default parameters. We used T-IDBA to assemble contigs with continuous k-mer value from 21 to 90 automatically and with other parameters set as default. Contigs generated by the above three software were then used to merge to the final non-redundant unigenes, using the TGICL with parameters set as default.

### Gene and gene family annotation

To annotate each of the six silkmoth transcriptomes, we performed a BLAST search against the non-redundant (NR) database in NCBI, SWISS-PROT, KEGG and COG with an e-value cut-off of 1e-5. We annotated the motifs and domains using InterPro. Gene ontology terms were assigned by Blast2GO through a search of the NR database. After sequence alignment, gene families were constructed and orthologous relationships were identified using TreeFam- 4.0.

### Phylogeny analysis

We reconstructed the phylogeny trees using coding sequences of 1342 single-copy-orthologs. Nucleotides of these genes were aligned by translated protein sequences. Three methods were used to reconstruct phylogenetic trees. 1) The Bayes tree was constructed by MrBayes-3.2.3 [50] with GTR + gamma substitution model. The generation number was set as 100000000 and the first 25% was set as burn-in. Other parameters were set as default; 2) the ML tree was constructed by maximum likelihood method with Tamura-Nei model; 3) the NJ tree was constructed by Neighbor-join method with Tajima-Nei model. Reconstruction of NJ tree and ML tree are generated by the software MEGA 5.0 [51].

### Analysis of molecular evolution of N termini of silk protein Fibroin H

N termini of silk protein Fibroin H orthologs were aligned by ClustalW [52]. To estimate dS and dN using PAML version 4.7, we first assumed the same dN/dS for all branches and estimated the ɷ values under one ratio model. Second, the ɷ values for all branches were calculated under the free ratio model. Then we performed likelihood ratio test to compare the fitness of these models with the data.

### Phylogenetic analysis on sericin genes

Silkworm sericin proteins, Ser-1, Ser-2 and Ser-3 were used to search for orthologs in the six silkmoths with via BLASTP, respectively. The best hit from each BLASTP search was selected for further analyses. maximum likelihood method with bootstrap replicates set as 1000. Then the gene tree was reconciled with the species tree by Notung 2.6 [53] with default parameters.

### Gene expression value measurement and comparison

For each unigenes, expression profiling was measured by mapping reads to assembled sequences using SOAP [54]. Then, the RPKM value for each transcript was measured in reads per kilobase of transcript sequence per million mapped read. RPKM of each family was used for comparison of expression level among the six silkmoths. RPKM of a family in a species was measured in reads per kilobase of all transcript sequences of all the members in that family per million mapped reads. To exclude the influence of evolutionary relationships on the identified specific gene families in colored-cocoon group and non-colored-cocoon group, we firstly evaluate the relationship between the color retention (we labeled “1” with stable coloration while “0” for unstable coloration) and each of gene families identified were evaluated based on correlation analyses, using phylogenetically independent contrasts. Gene families that didn’t show significant positive relationship with color retention were excluded from further analyses. Differential genes families between colored groups and non-colored groups using were identified by edgeR package in R project [55], with FDR < 0.05.

### Enrichment analysis

Enrichment analysis for the supplied gene list was carried out by online software WEGO [56] (http://wego.genomics.org.cn/cgi-bin/wego/index.pl), in which the p-value was approximated by the chi-square test.

### Accession codes

RNA-seq data have been deposited into the NCBI Short Read Archive (SRA, http://www.ncbi.nlm.nih.gov/sra/) under the accession number SRP050590 (SRX831712 - SRX831717). The transcriptome Shotgun Assemblies have been deposited at GenBank (TSA, http://www.ncbi.nlm.nih.gov/genbank/tsa) under the accession number GBZC00000000- GBZF00000000; GBZJ00000000 and GBZL00000000). The versions described in this paper are the first versions, i.e. GBZC01000000. GBZC01000000- GBZF01000000; GBZJ01000000; and GBZL01000000).

## Additional files

Additional file 1: Table S1. Summary of comparisons between assembled transcripts of the six silkmoths and the silkworm genes and transcripts. Additional file 2: Figure S1. GO terms for the transcriptomic sequences of the six silkmoths. **Figure S2.** COG categories for the transcriptomic sequences of the six silkmoths. **Figure S3.** Go enrichments of the six silkmoth specific genes. **Figure S4.** Phylogenetic trees constructed based concatenated alignment of single-copy proteins by maximum likelihood analyses (a) and Neighbor-join method (b), indicating similar topology. a) The ML tree was constructed by maximum likelihood method and bootstrap replicates set as 1000. b) The NJ tree was constructed by Neighbor-join method with Tajima-Nei model and Bootstrap replicates set as 1000. Bootstrap values were shown above the related node. **Figure S5.** Aligned sequences (a) and expression levels (b) of BBP orthologs. **Figure S6.** Aligned sequences (a) and expression levels (b) of CBP orthologs. Additional file 3: Data S1. Families identified by TreeFam of the six silkmoths and *B. mori*. Additional file 4: Data S2. Deduced amino acid sequences of Fibroin H repeat regions. Additional file 5: Data S3. Alignment of deduced amino acid sequences of Sericins.
