# Erratum to: Comparative transcriptome analyses on silk glands of six silkmoths imply the genetic basis of silk structure and coloration

**DOI:** 10.1186/s12864-017-3940-y

**Published:** 2017-07-21

**Authors:** Yang Dong, Fangying Dai, Yandong Ren, Hui Liu, Lei Chen, Pengcheng Yang, Yanqun Liu, Xin Li, Wen Wang, Hui Xiang

**Affiliations:** 1 Kunming University of Science and Technology, 727 South Jingming Road, Chenggong District, Kunming, Yunnan Province 650500 China; 2 State Key Laboratory of Genetic Resources and Evolution, Kunming Institute of Zoology, Chinese Academy of Sciences, 32 East Jiaochang Road, Kunming, Yunnan Province 650223 China; 3 State Key Laboratory of Silkworm Genome Biology, Key Sericultural Laboratory of Agricultural Ministry, Institute of Sericulture and Systems Biology, Southwest University, 2 Tiansheng Road, Beibei District, Chongqing, 400715 China; 4 Institute of Zoology, Chinese Academy of Sciences, 69 East Beichen Road, Chaoyang District, Beijing, 100101 China; 5 Shenyang Agricultural University, 120 Dongling Road, Shenhe District, Shenyang, Shenyang Province 110866 China; 6 Center for Epigenetics, Johns Hopkins University School of Medicine, Baltimore, MD 21205 USA

## Erratum

After the publication of this work [1], we were requested to make a supplementary note clarifying the sampling of the silkmoths used in our study, especially Antheraea assama. Larvae of A. pernyi, Samia Cynthia ricini and A. yamamai were provided by Shenyang Agricultural University, Hunan Nanlingence and technology development CO.LTD (https://hnnltckj.cn.china.cn), respectively. Larvae of Actias selene and Rhodinia newara was provided by Dr. Yun Wu, a specialist on ecology of butterflies and silk moths [2]. Antheraea assama, was collected in Xishuangbanna, Yunnan Province, China, and reared indoor with Dr. Yun Wu’s kind help. In his book [2], Dr. Wu also illustrated A. assama in Yunnan province, China. As to collection and rearing of this species, briefly, moths in the wild are collected by a light trap [2]. Pregnant female moths were then brought indoor and kept at room temperature. Eggs were laid in a container and collected and disinfected. The eggs were then hatched in incubator at 28 °C. The larvae were fed with fresh leaves with branches of a host plant Cinnamomum japonicum until the 5th instar, when it is ready for sampling. Antheraea assama is an important economic insect in India. These species also inhabits Yunnan province, China [2, 3]. Recently, Chinese researchers have been making efforts on artificial rearing this species and now it can be reared in laboratory condition [2, 4–6] (Additional file 1: Figure S1).

We regret any inconvenience that this inaccuracy might have caused.

## Additional file

Additional file 1: Figure S1. Antheraea assama in Yunnan province, China. A. Eggs and newly hatched larvae. B. The fifth instar larva. C. Male (left) and female (right) Pupae. D. Mating Adults. Cocoons (E) and female moths ready for laying eggs (F) are harvested by indoor rearing. All the photos were taken by Dr. Zhong J. Copyrights are asserted and protected ( 213 kb)
