# Supplementary material for: Comparative transcriptome analyses on silk glands of six silkmoths imply the genetic basis of silk structure and coloration
Source: BMC Genomics. 2015 Mar 17;16(1):203. doi: 10.1186/s12864-015-1420-9 (PMC4372302; doi:10.1186/s12864-015-1420-9)
Supplement: Additional file 1: Table S1. — Summary of comparisons between assembled transcripts of the six silkmoths and the silkworm genes and transcripts. [file 12864_2015_1420_MOESM1_ESM.doc]

**Table S1**. **Summary of comparisons between assembled transcripts of the six silkmoths and the silkworm genes and transcripts.**

|  | **Silkworm predicted genes (14,623)** |  | **Silkworm assembled transcripts (37,408)** |
| --- | --- | --- | --- |
| **A.ass (37,758)** | 10920  36940  11649 |  | 10209  8967 |
| **A.per (48,422)** | 47290 |  | 10758  10353 |
| **A.yam (46,948)** | 11539  45796 |  | 10555  10419 |
| **Ac.sel (48,053)** | 11724  46966  12046 |  | 10825  11399 |
| **R.new (51,734)** | 50811  11695 |  | 10856  11941  10561 |
| **S.cyn (47,632)** | 46541 |  | 10679 |

**Note1**: Numbers in the brackets refer to the numbers of transcripts / genes. For each grid, the number in the left upper refers to the number of matched hits in silkworm predicted genes/transcripts; the number in the lower right refers to the number of matched hits in the related silkmoth.

**Note2**: silkworm transcripts unmapped to the silkworm genome were used.
