# Supplementary material for: Comparative transcriptome analyses on silk glands of six silkmoths imply the genetic basis of silk structure and coloration
Source: BMC Genomics. 2015 Mar 17;16(1):203. doi: 10.1186/s12864-015-1420-9 (PMC4372302; doi:10.1186/s12864-015-1420-9)
Supplement: Additional file 2: Figure S1. — GO terms for the transcriptomic sequences of the six silkmoths. Figure S2. COG categories for the transcriptomic sequences of the six silkmoths. Figure S3. Go enrichments of the six silkmoth specific genes. Figure S4. Phylogenetic trees constructed based concatenated alignment of single-copy proteins by maximum likelihood analyses (a) and Neighbor-join method (b), indicating similar topology. a) The ML tree was constructed by maximum likelihood method and bootstrap replicates set as 1000. b) The NJ tree was constructed by Neighbor-join method with Tajima-Nei model and Bootstrap replicates set as 1000. Bootstrap values were shown above the related node. Figure S5. Aligned sequences (a) and expression levels (b) of BBP orthologs. Figure S6. Aligned sequences (a) and expression levels (b) of CBP orthologs. [file 12864_2015_1420_MOESM2_ESM.doc]

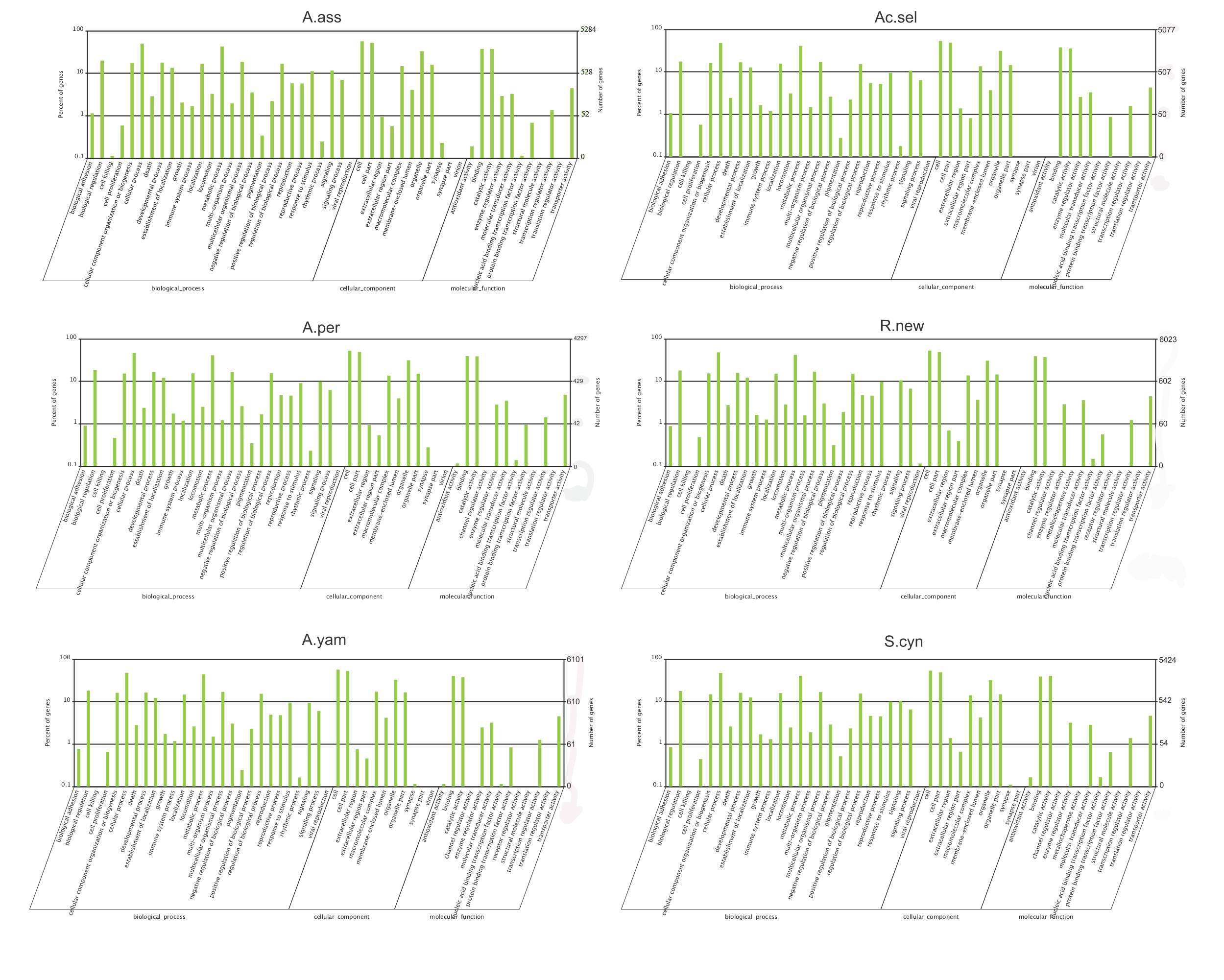


**Figure S1. GO terms for the transcriptomic sequences of the six silkmoths.**


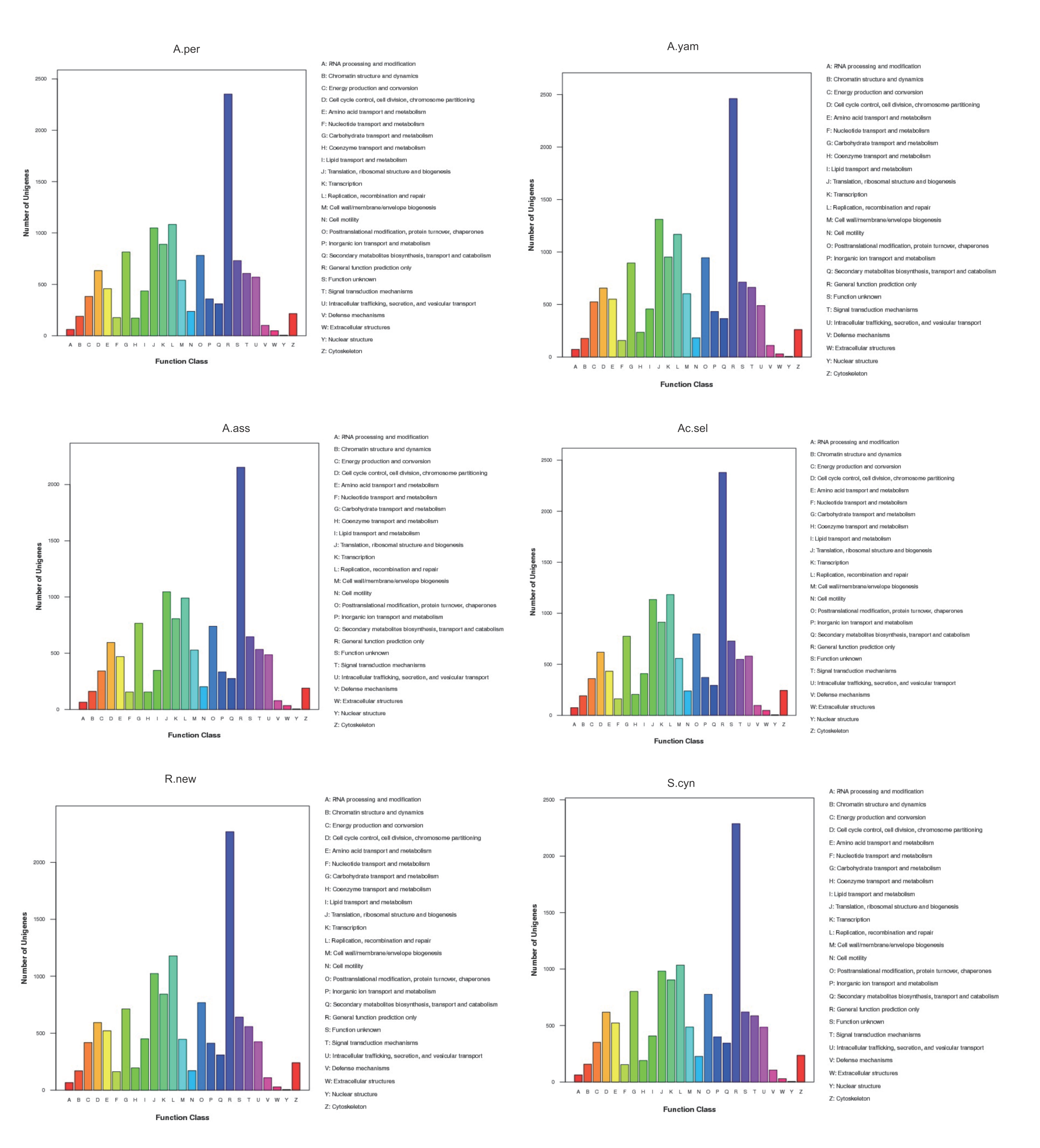


**Figure S2.** **COG categories for the transcriptomic sequences of the six silkmoths.**


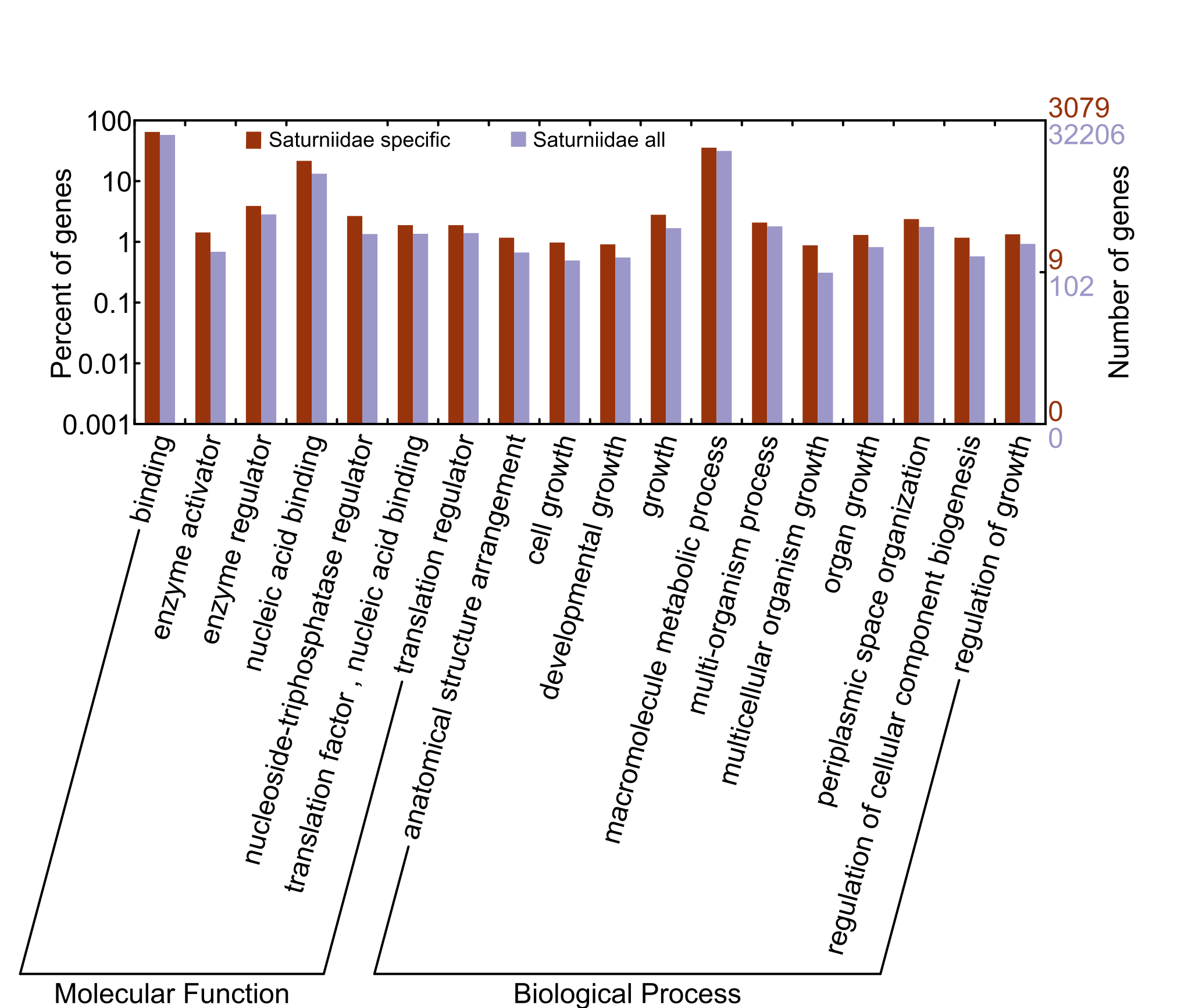


**Figure S3. Go enrichments of the six silkmoth specific genes.**


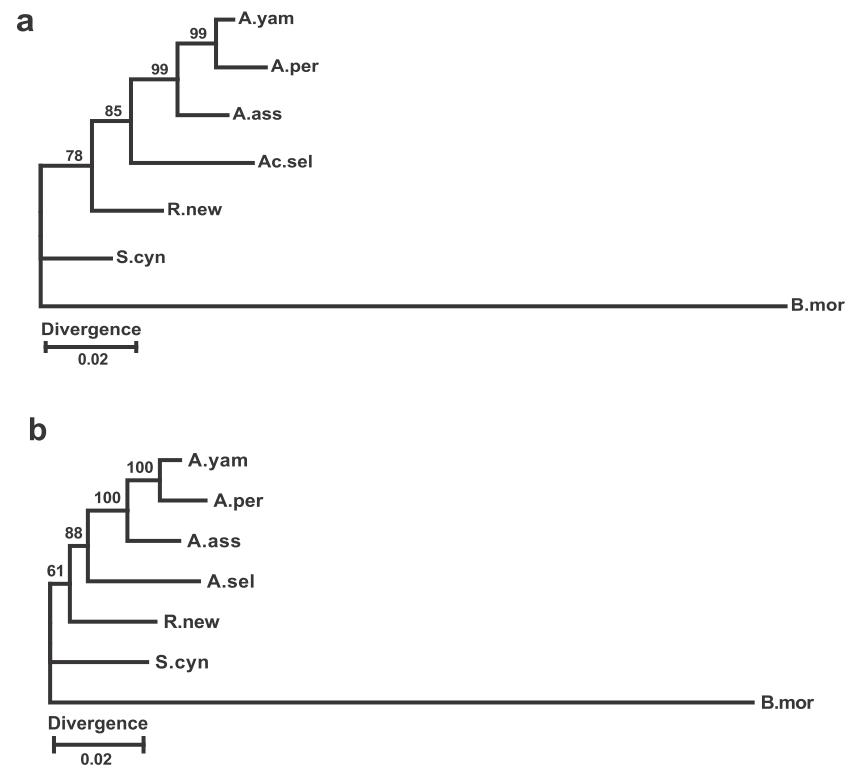


**Figure S4. Phylogenetic trees constructed based concatenated alignment of single-copy proteins by maximum likelihood analyses (a) and Neighbor-join method (b), indicating similar topology. a)** The ML tree was constructed by maximum likelihood method with Tamura-Nei model and bootstrap replicates set as 1000. b) The NJ tree was constructed by Neighbor-join method with Tajima-Nei model and Bootstrap replicates set as 1000. Bootrap values were shown above the related node.


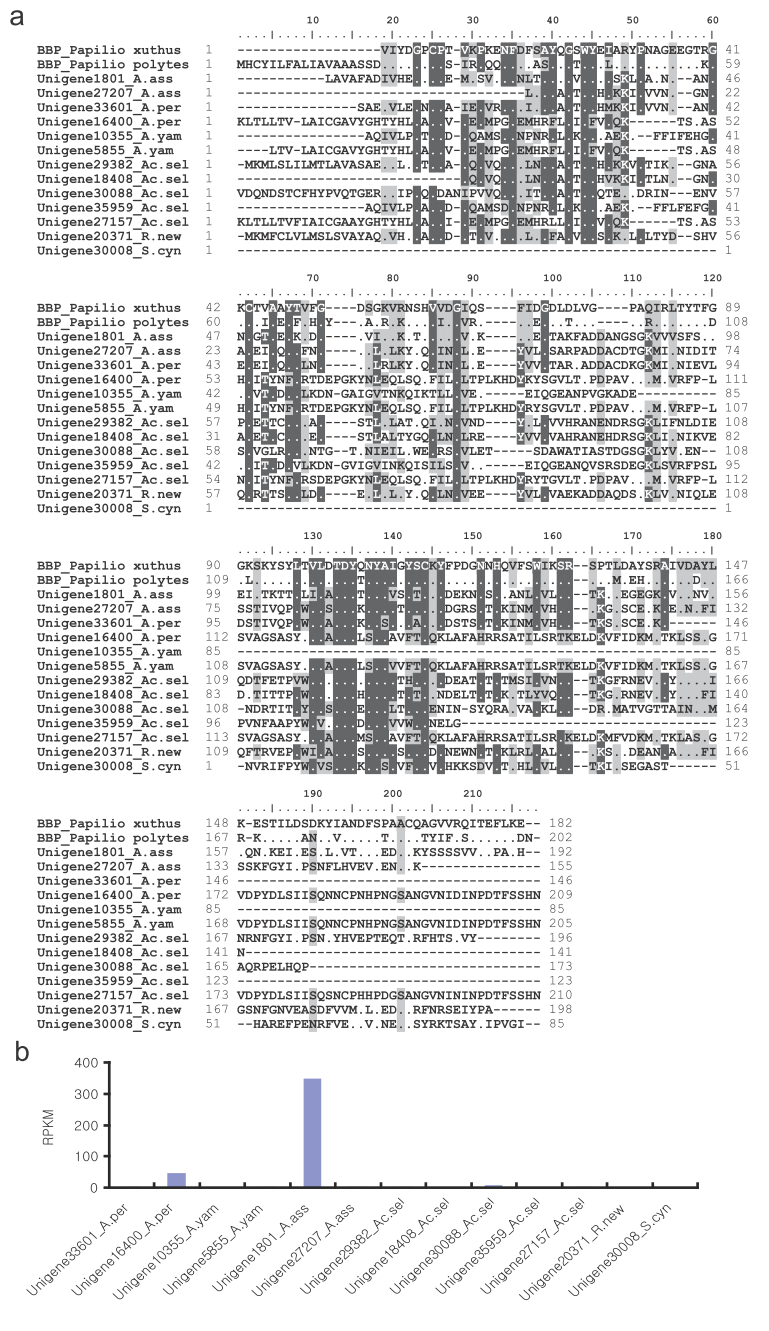


**Figure S5. Aligned sequences (a) and expression levels (b) of BBP orthologs.**


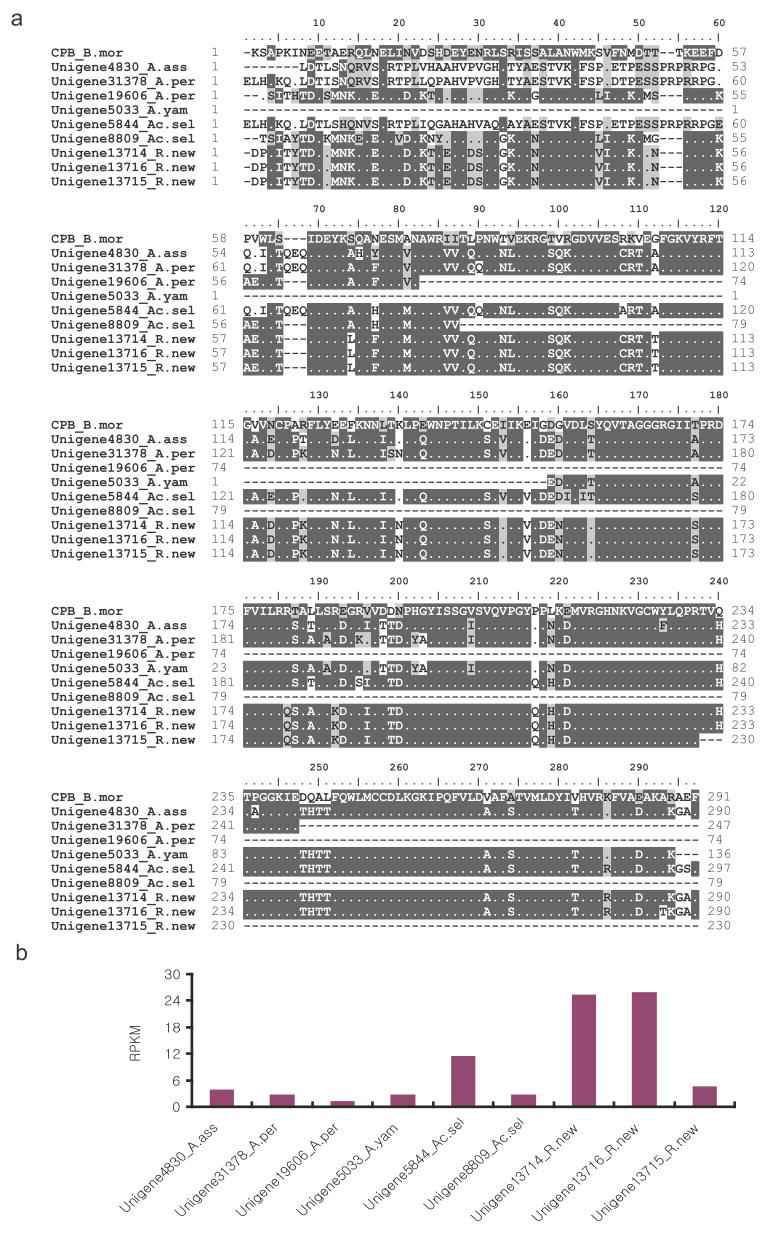


**Figure S6. Aligned sequences (a) and expression levels (b) of CBP orthologs.**
