# Supplementary material for: Erratum to: Comparative transcriptome analyses on silk glands of six silkmoths imply the genetic basis of silk structure and coloration
Source: BMC Genomics. 2017 Jul 21;18:548. doi: 10.1186/s12864-017-3940-y (PMC5520354; doi:10.1186/s12864-017-3940-y)

**Supplementary File 1**

**Caption:** Antheraea assama in Yunnan province, China. A. Eggs and newly hatched larvae. B. The fifth instar larva. C. Male (left) and female (right) Pupae. D. Mating Adults. Cocoons (E) and female moths ready for laying eggs (F) are harvested by indoor rearing. All the photos were taken by Dr. Zhong J. Copyrights are asserted and protected.


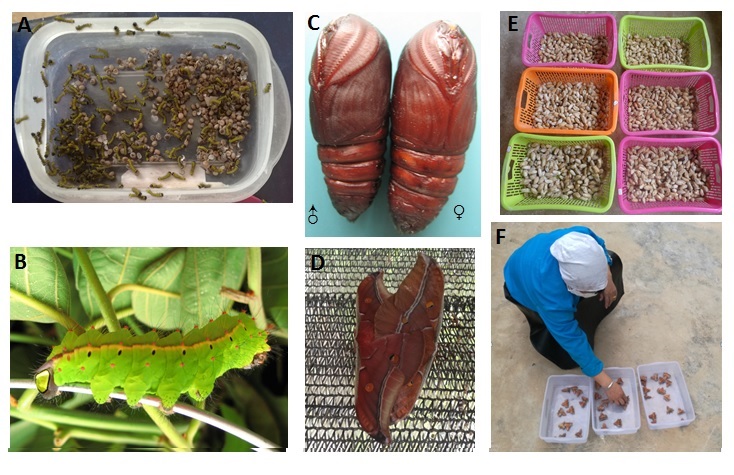

Supplement: Additional file 1: Figure S1. — Antheraea assama in Yunnan province, China. A. Eggs and newly hatched larvae. B. The fifth instar larva. C. Male (left) and female (right) Pupae. D. Mating Adults. Cocoons (E) and female moths ready for laying eggs (F) are harvested by indoor rearing. All the photos were taken by Dr. Zhong J. Copyrights are asserted and protected ( 213 kb) [file 12864_2017_3940_MOESM1_ESM.doc]
